# Magnetic Structure of Wiegand Wire Analyzed by First-Order Reversal Curves

**DOI:** 10.3390/ma15196951

**Published:** 2022-10-07

**Authors:** Liang Jiang, Chao Yang, Zenglu Song, Yasushi Takemura

**Affiliations:** 1 Department of Electrical and Computer Engineering, Yokohama National University, Yokohama 240-8501, Japan; 2 School of Electrical Engineering, Nanjing Vocational University of Industry Technology, Nanjing 210023, China

**Keywords:** Wiegand wire, magnetization reversal, large Barkhausen jump, first-order reversal curve (FORC), magnetic structure

## Abstract

Various coercive force field components in Wiegand wire exhibit a significant magnetization reversal under an applied magnetic field. A fast magnetization reversal is accompanied by a large Barkhausen jump, which induces a pulse voltage in a pickup coil wound around the Wiegand wire which serves as a power source for the devices or sensors. This study aims to elucidate the magnetization reversal in the Wiegand wire by using a first-order reversal curve (FORC) diagram method. The magnetic structure of the Wiegand wire typically comprises three layers: a soft layer, middle layer, and hard layer. In this study, we analyze the coercive and interactive force fields between the adjacent layers. The results demonstrate a high coercivity of the center core and a lower coercivity of the outer layer of the wire.

## 1. Introduction

Extensive research has been performed on the Wiegand effect since its discovery by John Wiegand in 1974 [1]. Vicalloy exhibits bistable characteristics under cyclic torsional strain and longitudinal strain, and the inner core and outer layer manifest various permanent magnetic properties, i.e., they exhibit varying coercivity [2]. The outer layer is a soft layer with low coercivity and the inner core is a hard layer with high coercivity. The magnetization direction of the soft layer reverses when the applied magnetic field is larger than the switching field, which depends on the minimum coercivity of the Wiegand wire, while the magnetization direction of the hard layer remains unchanged [3]. These variations in the coercivity of the outer layer and inner core lead to the magnetization direction of the inner core and outer layer periodically appearing as the same or opposite under the applied alternating magnetic field. If the magnetization direction of the inner core is opposite to that of the outer layer, it produces a large Barkhausen jump, which is also known as the “Wiegand effect” [4]. This differs from the amorphous metallic wires which can also generate the large Barkhausen jump that is caused by the random twisting stresses in the amorphous wires fabricated through in-rotating-water spinning technology [5,6,7,8]. In this context, Vicalloy is known as the Wiegand wire. A Wiegand sensor is created by winding the pickup coil on the Wiegand wire which produces a stable pulse voltage. The pulse width of the voltage is approximately 10 μs, which is close to 8 μs of the amorphous wire [6]. Furthermore, the generated voltage is unaffected by the frequency of the external magnetic field [4]. The operating temperature of Wiegand sensor ranges from approximately −200 °C to 180 °C and it exhibits a high signal/noise ratio [9]. This has led to the widespread usage of Wiegand wire in various research applications. Currently, Wiegand wire is widely used in rotary encoders and speed sensors, and an increasing number of studies are focused on improving its power generation performance.

The Wiegand sensor can be used to accumulate energy during magnetization reversal [10,11], and to drive the battery-less Hall sensor [12]. The pulse generated by the Wiegand wire can be utilized to provide power for medical implants [13]. The Wiegand sensor can also be employed in wireless energy transmission applications [14]. The Wiegand pulse also can be used in a linear positioning system [15]. Additionally, some studies have been conducted on the application circuit of the Wiegand sensor in recent years [16,17]. The aforementioned studies were primarily focused on the voltage and energy generated by the Wiegand wire in the pickup coil. Obtaining a better understanding of the magnetic structure and magnetization reversal process of the Wiegand wire presents considerable potential for the improvement in the output voltage and energy. For the voltage and energy, a uniform magnetization and complete magnetization reversal must exist during the large Barkhausen jump. The peak voltage and energy output of the Wiegand wire can be reduced by any variations in the magnetization direction [18].

Hitherto, only a few studies have been conducted on the magnetic structure and magnetization reversal. However, these studies were limited as they focused only on the pickup coil and hysteresis loop method. The coercive force field and interactive force field in each region of the Wiegand wire cannot be represented in detail [1,2]. The magnetic domain structure in Wiegand wire was determined by a pickup coil [19]. The magnetization reversal and magnetic structure of the Wiegand wire were analyzed using a first-order reversal curve (FORC) [20]. The outer layer of the Wiegand wire is the soft layer and the inner layer constitutes the hard core, which includes an interactive layer. However, these studies did not explain the coercive force field distribution of each layer in detail. The magnetic structure of Wiegand wires with varying lengths were analyzed by FORC and the coercive force distribution and interactive force were analyzed in detail [21]. Contrary to the aforementioned studies, certain researchers state that the outer layer of the Wiegand wire is the hard layer, whereas the inner layer forms the soft layer [22]. This study aims to thoroughly elucidate the magnetic structure of Wiegand wires with varying diameters and to analyze the distribution of the soft and hard layer. Additionally, the magnetization reversal process is evaluated. The major and minor hysteresis loops and FORC curves of the Wiegand wire are measured using a vibrating sample magnetometer (VSM).

We report the distribution of the coercive and interactive force fields in the Wiegand wire based on the wire diameter, which is evaluated in terms of the FORC diagram [20,21]. We conclusively demonstrate that the center core of the Wiegand wire is the hard magnetic part, whereas the outer layer is the soft layer exhibiting a lower coercive force. We also demonstrate that the magnetic structure of the Wiegand wire can be classified into three layers: soft layer, middle layer, and hard layer, which is a novel finding that has not been previously verified.

## 2. Materials and Methods

This study used Wiegand wires with a length of 13 mm and diameter of 0.23 mm, supplied from SWFE, Co. Ltd., Meishan, China. Their chemical composition was Fe_0.4_Co_0.5_V_0.1_, and their magnetization properties along with a large Barkhausen jump have been previously reported [4]. The fabrication of the wire can be summarized in four steps. Firstly, we apply sufficient tension in the length direction to the wire without stretching it. The tension is maintained during the 64 counterclockwise turns and 48 clockwise turns twist. Secondly, the wire is twisted counterclockwise and clockwise for eight and a half turns, which constitutes a cycle. After 17 cycles of twisting, the wire is twisted counterclockwise for eight and a half turns, which lasts for 10–15 s, and then slowly stretched by 1–2%. The counterclockwise and clockwise twisting is repeated in the second step for 60 cycles, maintaining tension without stretching the wire. Lastly, the wire is heated using a current of 5.6 A for 120 milliseconds, and the fabrication of the Wiegand wire is completed [23].

**Figure 1:**
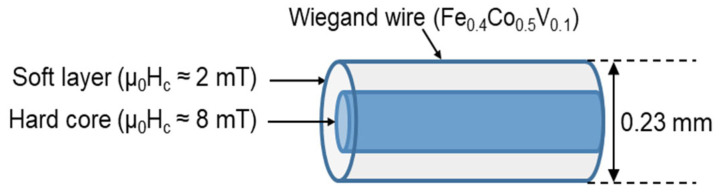
Magnetic structure of Wiegand wire.

Typically, the magnetic structure of the Wiegand wire comprises two layers, as shown in Figure 1. The outer layer is the soft layer with a coercivity of approximately 2 mT/μ_0_ and the inner layer is the hard core with a coercivity of approximately 8 mT/μ_0_; μ_0_ represents the permeability in vacuum. The coercivity varies gradually in the radial direction, implying the absence of any prominent boundary between the soft layer and the hard core [20]. FeCl_3_ solution was used to etch the outer surface of the Wiegand wire to further characterize the radial magnetic structure of the Wiegand wire [12]. The domain structure changed when the outer layers were etched. Therefore, we assumed that the wires also maintained the Wiegand effect, but the performance of the Wiegand effect changed. Four Wiegand wires with a length of 13 mm and diameter of 0.23 mm were placed horizontally in a glass beaker; a sufficient amount of FeCl_3_ solution was added to immerse the Wiegand wires. After a certain period (approximately 30 min), one Wiegand wire was removed and its diameter was measured in micrometers. If the diameter did not reach 0.18 mm, the Wiegand wire was placed back into the FeCl_3_ solution to continue etching for some more time. The Wiegand wire was then removed again and the diameter was measured. This process was repeated until the diameter of the Wiegand wire was 0.18 mm. Another Wiegand wire was removed approximately 20 min later, and the 0.14mm-diameter Wiegand wire was fabricated as described above. Subsequently, we fabricated the Wiegand wires with diameters of 0.10 mm and 0.06 mm following the same process. Thus, we prepared Wiegand wires with varying diameters of 0.06, 0.10, 0.14, 0.18, and 0.23 mm.

The FORC is an advanced tool that can be used to analyze the magnetic properties of magnetic materials [24]. The FORC can be used to directly obtain the coercive and interactive force distribution inside the magnetic particles, and present a detailed analysis of the types, sizes, and magnetic domain states of magnetic particles in various materials [25,26,27,28]. Therefore, it is very useful in determining the microscopic hysteresis characteristics of magnetic materials. The FORC method was first applied to analyze the magnetic structure of the Wiegand wire, and it was concluded that Wiegand wire can be segmented into the soft layer, middle layer, and hard core [20], which further demonstrated the effectiveness of the FORC method in analyzing the microscopic hysteresis characteristics of magnetic materials.

In this study, the major and minor hysteresis loop and FORC curve of the Wiegand wire were measured using VSM (Model 8600 series, Lake Shore Cryotronics, Westerville, OH, USA) at room temperature [20]. Additionally, the FORC diagram was derived using FORCinel and its auxiliary software (Igor Pro^®^, WaveMetrics Inc., Portland, OR, USA) [29]. The measurement process of the FORC curve employed in this study is as follows. Firstly, a saturated magnetic field, μ_0_H_sat_, was applied to render the Wiegand wire with positively saturated polarization. The applied magnetic field was then diminished to a reversal magnetic field, μ_0_H_a_. Consequently, the applied magnetic field increased to μ_0_H_sat_ from the reversal magnetic field, μ_0_H_a_. In this process, the magnetization curve of the Wiegand wire was defined as FORC. The FORC curve is dependent on the applied magnetic field and can be determined by the reversal magnetic field, μ_0_H_a_. By adjusting the reversal magnetic field, μ_0_H_a_, and repeating the process, a series of FORCs were obtained as μ_0_H_a_ varied from μ_0_H_sat_ to −μ_0_H_sat_ [30,31].

The max field of the major hysteresis loops was 1000 mT, and the steps of the applied magnetic field were 1 mT. The applied magnetic field of the minor hysteresis loops varied from 2 mT to 15 mT, and the steps of each applied magnetic field were 0.025 mT. The average measurement time of the major and minor hysteresis loops was 0.1 s. A magnetic field of μ_0_H_a_ = −500 mT to μ_0_H_sat_ = 500 mT was applied to measure the reversal curves of the Wiegand wires with varying diameters, and the steps of the reversal magnetic field, μ_0_H_a_, of each FORC curve were 0.5 mT [20]. The average measurement time of FORC was 0.1 s, and the number of FORCs was 121. The maximum coercive field was 30 mT, and the extreme value of interaction field was ±15 mT. The pause at the calibration field and the reverse fields was 1 s, and at the saturation field was 0.1 s.

**Figure 2:**
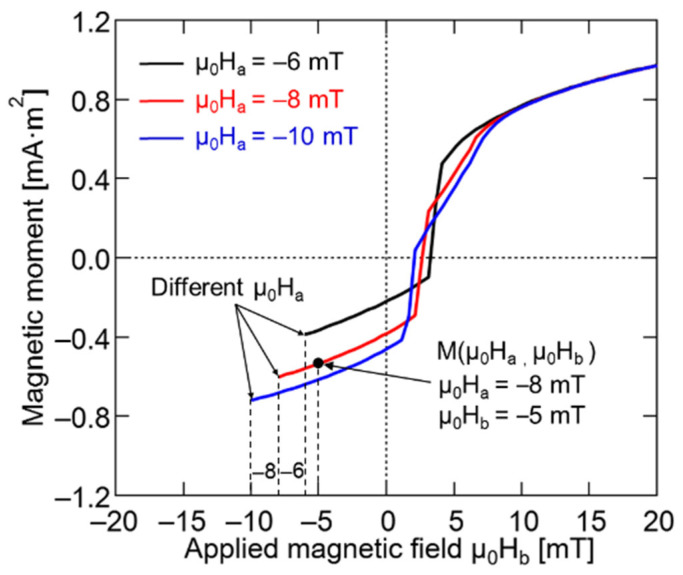
FORCs of Wiegand wire at various values of μ_0_H_a_. At the applied magnetic fields, (μ_0_H_b_) with reversal field, (μ_0_H_a_), black point of magnetization on FORC can be represented using M (μ_0_H_a_ = −8 mT, μ_0_H_b_ = −5 mT).

The magnetization intensity corresponding to any external magnetic field (μ_0_H_b_) on FORC can be expressed as M (μ_0_H_a_, μ_0_H_b_), where μ_0_H_b_ ≥ μ_0_H_a_, as shown in Figure 2. Here, the μ_0_H_a_ of the three FORC curves was −6 mT, −8 mT, and −10 mT, respectively, and the black point of magnetization on the FORC is represented by M (μ_0_H_a_ = −8 mT, μ_0_H_b_ = −5 mT). The distribution function, ρ (μ_0_H_a_, μ_0_H_b_), can be obtained based on the second derivative of all the measured M (μ_0_H_a_, μ_0_H_b_) values corresponding to μ_0_H_a_ and μ_0_H_b_. Thus, the distribution of M (μ_0_H_a_, μ_0_H_b_) on the (μ_0_H_a_, μ_0_H_b_) plane can be calculated using Equation (1), which yields the FORC diagram [32].

(1) $$\rho \left(\mu_{0}H_{a},\;\mu_{0}H_{b}\right)=-\frac{\partial^{2}M\left(\mu_{0}H_{a},{\;\mu }_{0}H_{b}\right)}{\partial \mu_{0}H_{a}{\;\mu }_{0}H_{b}}$$

The coordinate axis (μ_0_H_a_, μ_0_H_b_) must be converted to (μ_0_H_c_, μ_0_H_u_) to directly analyze the distribution of the coercive and interactive force fields in each region of the Wiegand wire, where μ_0_H_c_ and μ_0_H_u_ represent the coercive and interactive force fields, respectively. The conversion relation is expressed as follows:

(2) $$\mu_{0}H_{c}=(\mu_{0}H_{b}-\mu_{0}H_{a})/2,$$

(3) $$\mu_{0}H_{u}=(\mu_{0}H_{b}+\mu_{0}H_{a})/2$$

**Figure 3:**
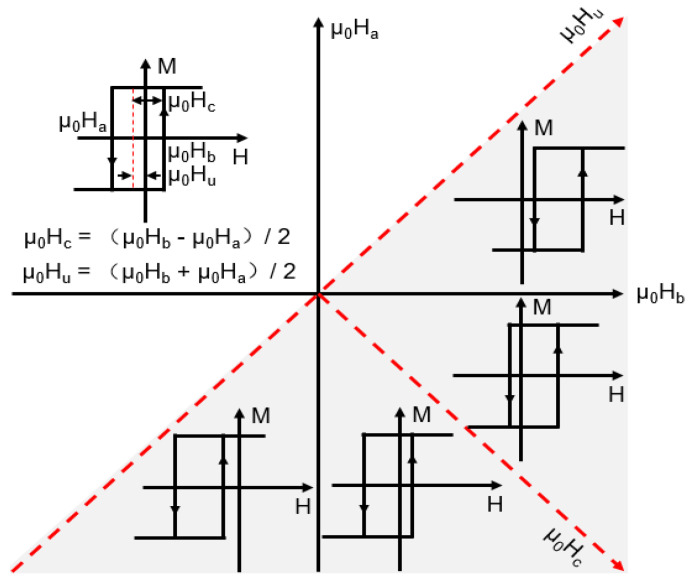
Coordinate composed of μ_0_H_a_ and μ_0_H_b_ or μ_0_H_c_ and μ_0_H_u_.

In the (μ_0_H_a_, μ_0_H_b_) coordinate system, the geometric interpretation of the distribution function, ρ (μ_0_H_a_, μ_0_H_b_), formulates the FORC density contour map represented by the gray triangle region (defined by ±μ_0_H_sat_) in Figure 3 [32]. The FORC density contour map in the coordinate system (μ_0_H_c_, μ_0_H_u_) can be obtained from the transformation relations expressed in Equations (2) and (3), as shown by the coordinate system of the red dotted line in Figure 3.

## 3. Results

### 3.1. Major Hysteresis Loops

**Figure 4:**
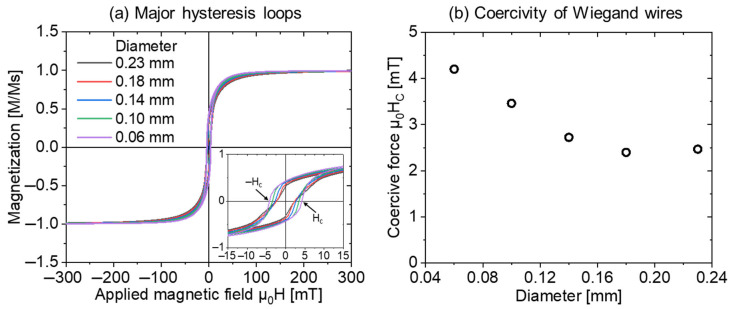
(**a**) Major hysteresis loops of Wiegand wires and (**b**) coercivity of Wiegand wires with varying diameters.

Figure 4a depicts the normal and magnified perspectives of the major hysteresis loops of five Wiegand wires with varying diameters. All the major hysteresis loops were normalized, and the magnified perspective clearly demonstrates that the coercivity of the Wiegand wires changed with varying diameters. Figure 4b depicts this variation trend. Therefore, the coercivity of Wiegand wire decreased with the increase in the diameter until it saturated at approximately 2.47 mT.

### 3.2. FORC Curves and FORC Diagrams

**Figure 5:**
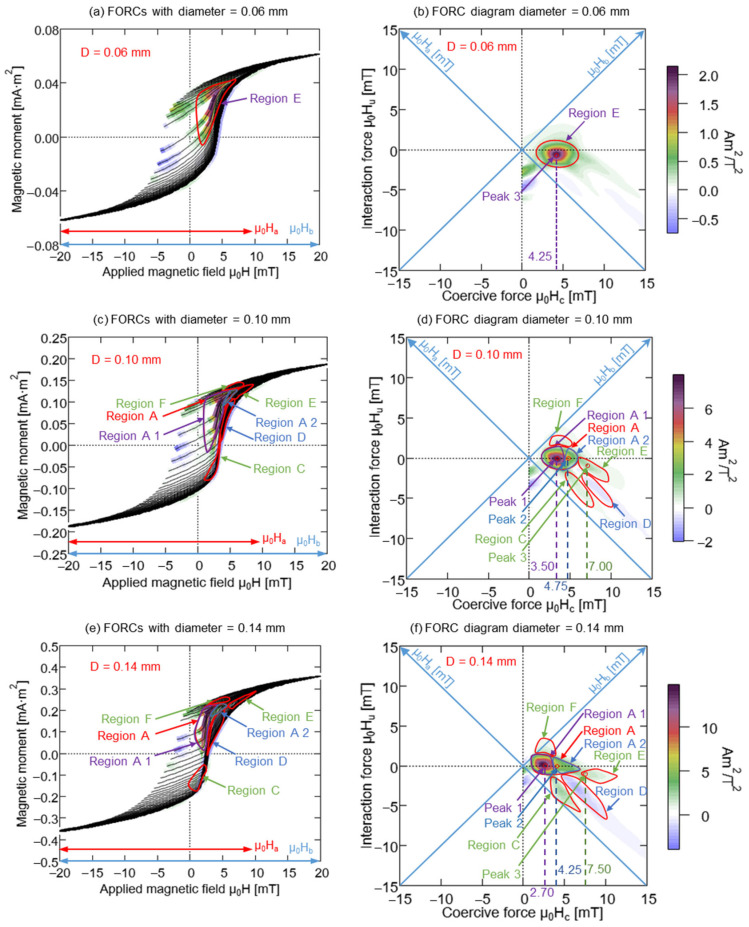

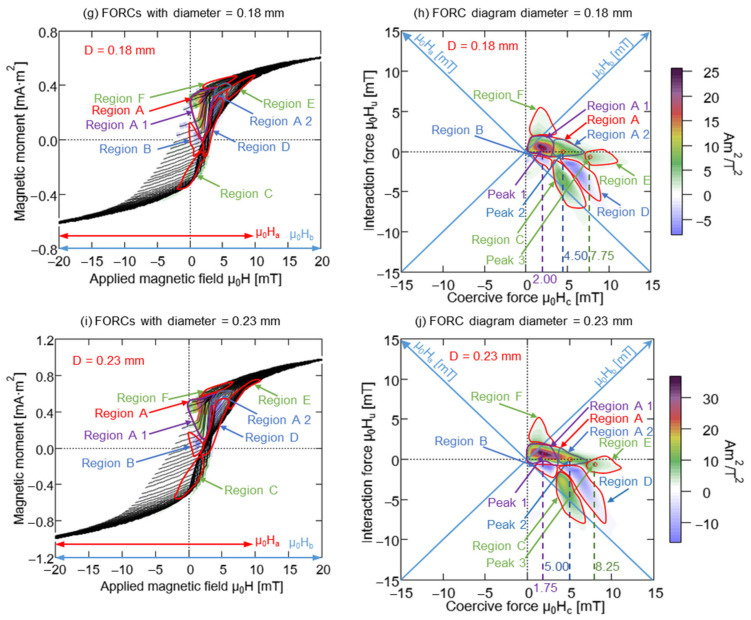
FORCs and FORC diagram of Wiegand wires with varying diameters.

Figure 5 presents the measurement results of the FORC curves of the Wiegand wires with varying diameters. The FORC curves between −20 mT and 20 mT are selected here because the irreversible magnetization curves considered in this study are all located within this range. The FORC diagrams of the Wiegand wires with varying diameters were calculated using FORC curves of corresponding diameters. The FORC curves illustrated in Figure 5a,c,e,g,i correspond to the Wiegand wires with diameters of 0.06, 0.10, 0.14, 0.18, and 0.23 mm, respectively. The FORC diagrams of the Wiegand wires with diameters of 0.06, 0.10, 0.14, 0.18, and 0.23 mm are plotted in Figure 5b,d,f,h,j, respectively. The horizontal and vertical axes of the FORC diagrams denote μ_0_H_c_ and μ_0_H_u_, respectively.

## 4. Discussion

### 4.1. Correlation between Coercivity of Major Hysteresis Loop and FORC Distribution of Wiegand Wire

**Figure 6:**
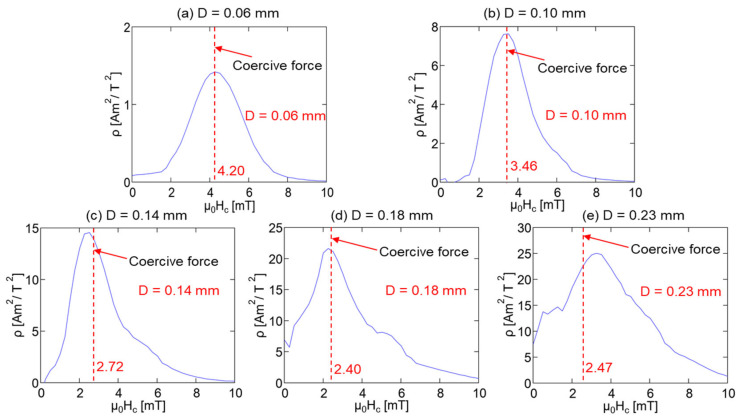
Correlation between maximum of FORC distribution on μ_0_H_c_ axis and coercivity of major hysteresis loop.

In Figure 5b,d,f,h,j for μ_0_H_u_ = 0 mT, the FORC distribution, ρ, is plotted along the axis of μ_0_H_c_, which is represented by the blue lines in Figure 6. It can be observed that the intensity of the FORC distribution, ρ, increased with the diameter of the Wiegand wire, whereas the coercivity, μ_0_H_c_, corresponding to the maximum intensity of the FORC distribution, ρ, gradually decreased and eventually tended to be constant. The coercive forces of the major hysteresis loops of the Wiegand wire are plotted in Figure 4b, which are represented by the red dotted lines in Figure 6. The comparative analysis demonstrated that the coercive force of the major hysteresis loop of the Wiegand wire with equal diameter was consistent with the maximum intensity of the FORC distribution, ρ [21].

### 4.2. Single and Uniform Magnetic Structure

**Figure 7:**
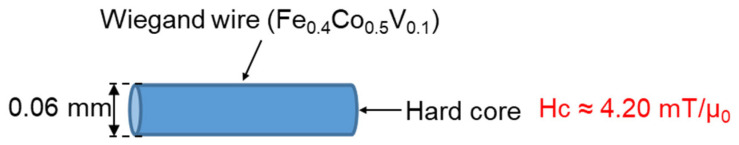
Single magnetic structure diagram.

The measured FORC diagram elucidates the magnetization process and magnetic interaction inside the Wiegand wire [33,34,35]. The FORC curve of the Wiegand wire with a diameter of 0.06 mm did not exhibit any prominent large Barkhausen jumps, as observed from Figure 5a. This indicates that the etched outer layer causes the fast magnetization reversal [12]. The various coercivity components in the same magnetic material can be analyzed in the FORC diagram. Furthermore, the FORC diagram presented in Figure 5b depicts only one region and a single peak, which indicates the existence of only one coercivity component. Its interactive field distribution was located close to μ_0_H_u_ = 0 mT, which can be considered a single and uniform magnetic structure. The coercivity of the 0.06 mm-diameter Wiegand wire was approximately 4.20 mT, which is greater than 2 mT and indicating that this single area is the hard core, as observed from Figure 4b. This further confirms that the inner layer of the Wiegand wire is the hard core and the outer layer is the soft layer. Interestingly, Peak 3 exhibited the maximum value of the FORC distribution and was located at the position of coercivity at ~4.25 mT, as observed from Figure 5b. This concurs with the coercivity of the 0.06 mm-diameter Wiegand wire. This single magnetic structure is depicted in Figure 7.

### 4.3. Three Layers of Magnetic Structure

According to the magnetic structure of the 0.06 mm-diameter Wiegand wire, the inner layer was the hard layer and the outer layer was the soft layer. We analyzed the FORC diagrams of the Wiegand wires with diameters of 0.10, 0.14, 0.18, and 0.23 mm, and observed that their FORC diagrams were segmented into six or seven small regions with similar distributions, implying the existence of more than one coercivity component [21]. The distribution intensity of each small area of the FORC diagram increased with the increase in the diameter, as stated in Section 4.1. These small regions are no longer distributed around the interactive force, μ_0_H_u_ = 0, which was similar to that of the 0.06 mm-diameter Wiegand wire; however, they expanded along the μ_0_H_u_ axis. The interactions between the components of the soft and hard layers within the Wiegand wire may cause these components to deviate from the zero-offset axis, which results in the formation of ridges [36]. It indicates an interaction between the soft and hard layers of the Wiegand wire, i.e., it indicates the existence of an interaction layer or middle layer.

It can be observed from Figure 5d,f,h,j that region A was distributed along the μ_0_H_c_ axis with a wide distribution range. In Figure 5c,e,g,i, region A was divided into the A1 and A2 regions. The A1 region presents a considerably large Barkhausen jump and the slope of the FORC curve is relatively steep. However, no large Barkhausen jump was observed in the A2 region, and the slope of the FORC curve was relatively flat. In Figure 5d,f,h,j, the A1 and A2 regions corresponding to Figure 5c,e,g,i were further segmented. Particularly, the distribution range of the coercive force in A1 was 0–4.25 mT, the coercivity was relatively small, and the absolute value of interactive force was less than 1.5 mT. Therefore, A1 can be considered as a soft layer with relatively small interaction.

In the FORC curve, the E region was primarily distributed in the reversible magnetization area and was situated farther away from the large Barkhausen jump. Additionally, the coercive force distribution ranged from 5.75 to 11.25 mT, and the absolute value of the interactive force was less than 2.75 mT. Therefore, E can be considered as a hard core with minimal interaction.

In A2, the coercive force distribution ranged from 3.25 to 6.5 mT, which lies between the soft layer and hard core, and the absolute value of the interactive force was less than 1.5 mT. Thus, A2 can be considered as a middle layer with relatively small interaction.

The interactive forces in regions C, D, and F were relatively large, and the maximum absolute value could reach 7.5 mT. Therefore, it can be assumed that these three regions together constitute the interaction layer and were formed by the interaction between the soft and hard layers of the Wiegand wire.

Region B was distributed prior to the large Barkhausen jump, as shown in Figure 5g,i. The distribution value of region B was negative, the distribution range of the coercive force was 0.75–3.25 mT, and the distribution range of the interactive force was −2 to −0.75 mT, as shown in Figure 5h,j. Therefore, region B can be considered as a soft layer with fewer interactions. If the applied magnetic field was 0–2.5 mT, the magnetization direction of a portion of the soft layer was opposite to that of the hard layer before the occurrence of the large Barkhausen jump, owing to the presence of the magnetostatic coupling and the demagnetizing field. This indicates the existence of negative region B. Region B in the soft layer was gradually etched away with the decrease in the diameter, and is not present in Figure 5c,e.

**Figure 8:**
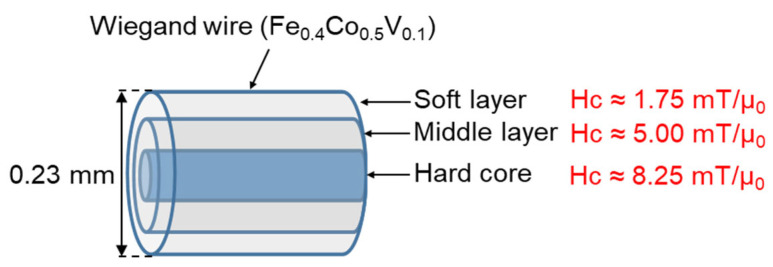
Magnetic structure diagram of Wiegand wire with 0.23 mm diameter.

**Figure 9:**
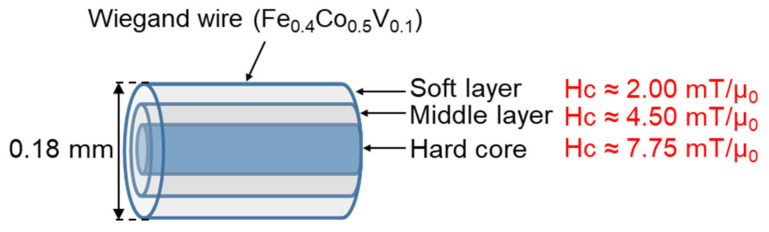
Magnetic structure diagram of 0.18 mm-diameter Wiegand wire.

**Figure 10:**
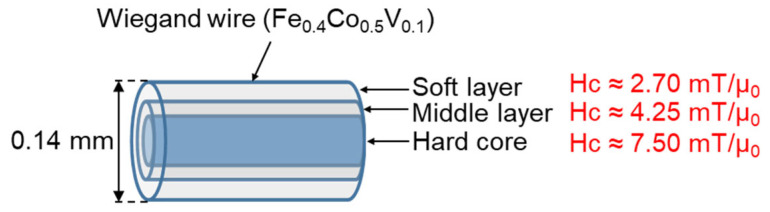
Magnetic structure diagram of 0.14 mm-diameter Wiegand wire.

**Figure 11:**
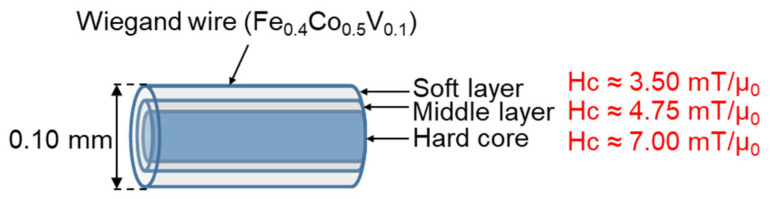
Magnetic structure diagram of 0.10 mm-diameter Wiegand wire.

According to the analysis conducted in Section 4.1, the coercive force of the Wiegand wire concurs with the maximum value of the FORC distribution, ρ. Therefore, the coercivity of the soft, middle, and hard layers could be obtained from Figure 5d,f,h,j, respectively. Furthermore, the coercivity of the soft and hard layers of the 0.23 mm-diameter Wiegand wire is consistent with the findings of previous studies [20]. Figure 8, Figure 9, Figure 10 and Figure 11 depict their magnetic structures, and Table 1 lists the FORC data.

A comparison of the magnetic structures of five Wiegand wires with varying diameters demonstrates that the coercivity of the hard core increased with the inclusion of the soft layer. This coercivity gradually increased with the increase in the volume of the soft layer, which further established the interaction between the soft and hard layers. This is consistent with the findings of previous studies [20].

### 4.4. Relationship between Magnetization Reversal Direction of Wiegand Wire and Partial Region of FORC Distribution Diagram

**Figure 12:**
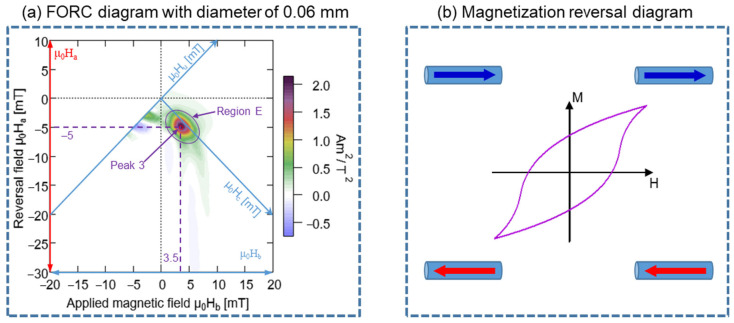
(**a**) FORC diagram of 0.06 mm-diameter Wiegand wire. (**b**) Magnetization reversal diagram of minor loop.

According to the analysis conducted in Section 4.2, the 0.06 mm-diameter Wiegand wire comprises a single and uniform magnetic structure, which corresponds to the hard core of the Wiegand wire. Figure 12a depicts its FORC distribution with μ_0_H_b_ as the horizontal axis and μ_0_H_a_ as the vertical axis. The value of the magnetization reverse field and the applied magnetic field at each point of the FORC distribution can be analyzed in detail. For instance, the maximum point of the FORC distribution, Peak 3, corresponds to the magnetization reverse field, μ_0_H_a_ = −5 mT, and the applied magnetic field, μ_0_H_b_ = 3.5 mT. In the figure, only region E presents a prominent distribution intensity, whereas that of the other regions is typically zero. The minor hysteresis loop of the 0.06 mm-diameter Wiegand wire is presented in Figure 12, which reflects a smooth hysteresis loop. During the magnetization process of the Wiegand wire, no prominent large Barkhausen jump was observed, and only two magnetization directions could be detected. One is represented by the blue arrow on the right-hand side and the other is represented by the red arrow on the left-hand side.

**Figure 13:**
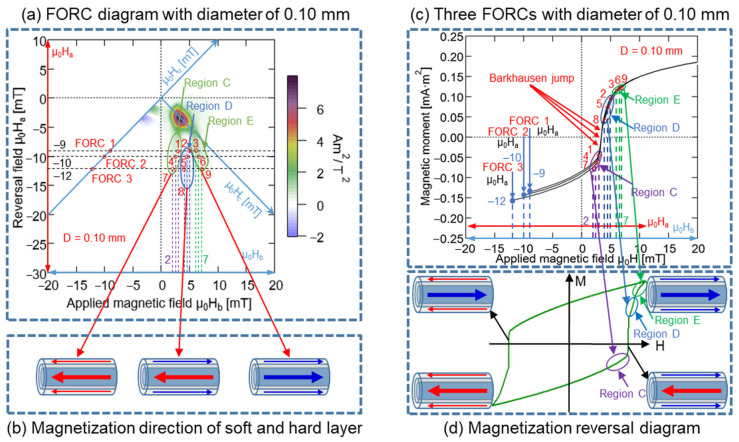
(**a**) FORC diagram of 0.10 mm-diameter Wiegand wire; three FORCs with varying reversal fields μ_0_H_a_ of −9, −10, and −12 mT. (**b**) Magnetization reversal direction of soft and hard layer. (**c**) Three FORCs for 0.10 mm-diameter Wiegand wire under reversal fields of −9, −10, and −12 mT. (**d**) Magnetization reversal diagram of minor loop.

The analysis of the FORC curve and diagram of the 0.10mm-diameter Wiegand wire was presented as an example [21]. In Figure 13a, μ_0_H_b_ denotes the horizontal axis and μ_0_H_a_ represents the vertical axis. According to the previous analysis, region C was primarily distributed in the region located prior to the large Barkhausen jump, region D was primarily distributed in the region after the large Barkhausen jump, and region E was primarily distributed in the region situated farther away from the large Barkhausen jump. Particularly, three FORC curves were selected with μ_0_H_a_ equal to −9, −10, and −12 mT: FORC1, FORC2, and FORC3. All three FORC curves passed through regions, C, D, and E, wherein a total of nine points were considered from the three FORC curves with the coordinates of 1 (−9, 3), 2 (−9, 5), 3 (−9, 6), 4 (−10, 2.5), 5 (−10, 4.5), 6 (−10, 6.5), 7 (−12, 2), 8 (−12, 4), and 9 (−12, 7), respectively. Overall, points 1, 4, and 7 were distributed in region C; points 2, 5, and 8 were distributed in region D; and points 3, 6, and 9 were distributed in region E.

**Figure 14:**
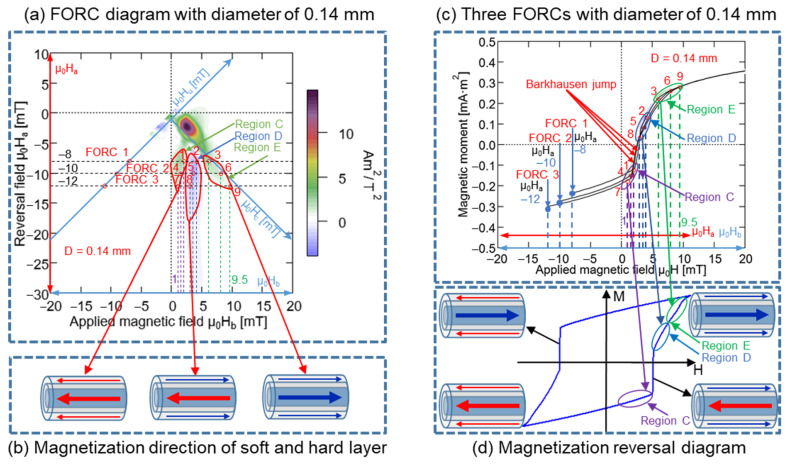
(**a**) FORC diagram of 0.14 mm-diameter Wiegand wire; three FORCs with varying reversal fields, μ_0_H_a_, of −8, −10, and −12 mT, respectively. (**b**) Magnetization reversal direction of soft and hard layer. (**c**) Three FORCs with 0.14 mm-diameter under reversal fields of −8, −10, and −12 mT. (**d**) Magnetization reversal diagram of minor loop.

**Figure 15:**
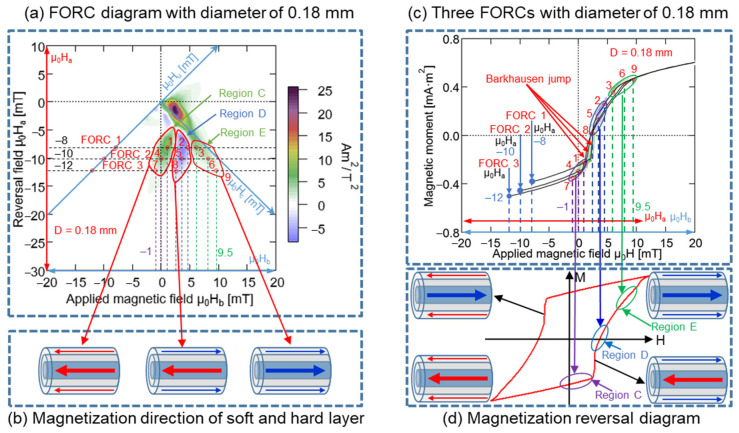
(**a**) FORC diagram for 0.18 mm-diameter Wiegand wire; three FORCs for varying reversal fields μ_0_H_a_ of −8, −10, and −12 mT. (**b**) Magnetization reversal direction of soft and hard layer. (**c**) Three FORCs for 0.18 mm-diameter Wiegand wire for reversal fields of −8, −10, and −12 mT. (**d**) Magnetization reversal diagram of minor loop.

**Figure 16:**
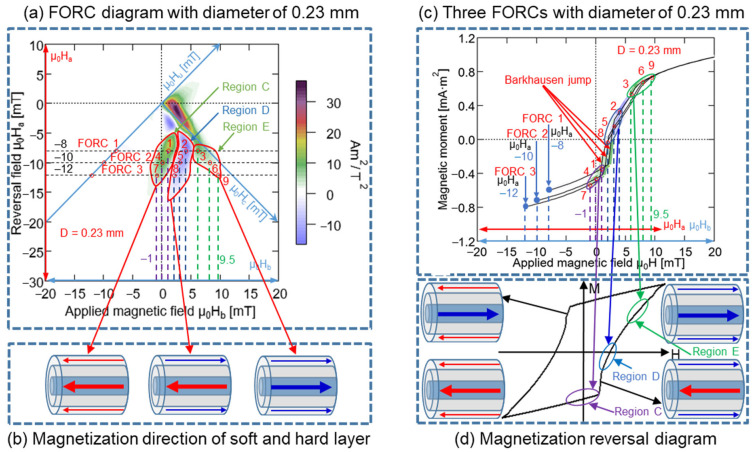
(**a**) FORC diagram for 0.23 mm-diameter Wiegand wire, three FORCs with varying reversal fields μ_0_H_a_ of −8, −10, and −12 mT. (**b**) Magnetization reversal direction of soft and hard layer. (**c**) Three FORCs for 0.23 mm-diameter Wiegand wire in reversal fields of −8, −10, and −12 mT. (**d**) Magnetization reversal diagram of minor loop.

In the FORC curves presented in Figure 13c, these nine points and the three regions, C, D, and E, respectively, were marked. A comparison of Figure 13c,d demonstrated that certain regions of the minor hysteresis loop in Figure 13d correspond to the regions C, D, and E in Figure 13c. Region C was distributed before the large Barkhausen jump, and same magnetization direction remained unchanged in the soft and hard layers of the Wiegand wire, i.e., toward the left-hand side. Conversely, region D was distributed after the large Barkhausen jump, and the opposite magnetization direction was observed for the soft and hard layer of Wiegand wire, i.e., the soft layer was oriented toward the right-hand side and the hard layer was oriented toward the left-hand side. Although Region E was distributed after the large Barkhausen jump, the magnetization direction remained unchanged over the soft and hard layers, i.e., toward the right-hand side. The three magnetization states in Figure 13b correspond to the three regions in Figure 13a, respectively. Therefore, the FORC diagram was positive for the same magnetization directions of the soft and hard layers, whereas it was negative for opposite magnetization directions of the soft and hard layers. The negative region appearing in the FORC diagram of the Wiegand wire was generated by the interaction between the soft and hard layers [20]. The analysis method employed for the Wiegand wires with diameters of 0.10 mm was applied for those of 0.14, 0.18, and 0.23 mm. Particularly, three FORC curves with μ_0_H_a_ of −8, −10, and −12 mT were selected, and the results are presented in Figure 14, Figure 15 and Figure 16. The same conclusion inferred for the Wiegand wire with a diameter of 0.10 mm can be derived for the Wiegand wires with diameters of 0.14, 0.18, and 0.23 mm. That is, the FORC diagram is positive for the same magnetization directions of the soft and hard layers, and it is negative for opposite magnetization directions of the soft and hard layers.

## 5. Conclusions

In this study, the major and minor hysteresis loops and FORC curves of five samples were measured using VSM, and the corresponding FORC diagrams were obtained. According to the analysis of the measurement data of five samples, if the adequate volume is etched away from the outer layer of the Wiegand wire, only a single uniform magnetic structure with coercivity of 4.20 mT remains. Therefore, the central core is the hard magnetic component. Particularly, the inner layer of the Wiegand wire is the hard layer and the outer layer is the soft layer. We analyzed the coercive force field and interactive force field of the FORC diagram of the Wiegand wires with varying diameters. We observed that there was no evident boundary between the soft and hard layers of the Wiegand wires. However, an interaction exists between the soft and hard layers along with a corresponding middle layer, i.e., the magnetic structure of the Wiegand wire consists of three layers. The interaction between the hard core with a coercivity of 4.20 mT and the soft layer increases the coercivity of the hard core. The coercivity of the hard core increases with the increase in the volume of the soft layer. That is, the coercivity of the hard core increases with the increase in the interaction between the hard core and the soft layer. The comprehensive coercivity of the magnetic material was produced by the combined action of all the coercivity components. The overall coercivity of the Wiegand wire decreases if the volume of the soft layer with low coercivity increases. When the outer layers of the Wiegand wires were etched, the domain structure and the spatial distribution of the magnetization changed, but we assumed that the wires also retained the Wiegand effect. Therefore, we can determine the coercive force distribution of the soft, middle, and hard layer with varying diameters, which presents considerable potential for the analysis of the magnetization reversal characteristics of the Wiegand wire. These features help in further improving the output voltage and energy derived from the Wiegand wire for self-powered devices and sensors.

## Figures and Tables

**Table 1:** Distribution of coercivity and interaction fields in various regions of Wiegand wires with varying diameters.

Region or Peak	D = 0.06 mm		D = 0.10 mm		D = 0.14 mm		D = 0.18 mm		D = 0.23 mm		Attribute
	μ_0_H_c_ (mT)	μ_0_H_u_ (mT)	μ_0_H_c_ (mT)	μ_0_H_u_ (mT)	μ_0_H_c_ (mT)	μ_0_H_u_ (mT)	μ_0_H_c_ (mT)	μ_0_H_u_ (mT)	μ_0_H_c_ (mT)	μ_0_H_u_ (mT)	
Region A1	\	\	1.75–4.25	−1.5 to 1.25	1.25–3.5	−1 to 1.25	0.25–3.25	−0.75 to 1.5	0–3.5	−0.5 to 1.5	Soft layer
Region A2	\	\	4.25–5.5	−0.75 to 0.75	3.5–6.5	−0.75 to 0	3.25–6.5	−0.75 to 1.25	3.5–6.5	−0.5 to 1.25	Middle layer
Region B	\	\	\	\	\	\	0.75–3	−2.25 to −0.75	0.75–3.25	−2 to −0.75	Soft layer
Region C	\	\	4.5–7.5	−5.5 to −1.5	3.5–6.5	−5.5 to −1.5	3.25–6.5	−6.5 to −1.25	3.25–6.5	−7.5 to −1.5	Interaction
Region D	\	\	6.5–10	−5.5 to −2	4.5–9.75	−6.75 to −1	4–8.25	−5.75 to −1	4–9	−7.5 to −1	Interaction
Region E	2.25–6.5	−2 to 1.25	5.75–9.75	−2.75 to 0.25	6.25–11.25	−2.25 to −0.25	6.5–10.5	−2 to 0.25	6.5–11.25	−2 to 0.5	Hard core
Region F	\	\	2–5.5	1–3	1–3.75	1–3	0.25–3.5	1.5–4.75	0.25–2.5	1.25–5	Interaction
Peak 1	\	\	3.5	0	2.75	0.25	2	0.5	1.75	0.75	Soft layer
Peak 2	\	\	4.5	0	3.75	−0.25	3.5	0	4.25	0.25	Middle layer
Peak 3	4.25	−0.75	7	−1	7.5	−1	7.75	−0.75	8.25	−0.75	Hard core

## Data Availability

Not applicable.
